# Benefit of cervical screening at different ages: evidence from the UK audit of screening histories

**DOI:** 10.1038/sj.bjc.6600974

**Published:** 2003-07-01

**Authors:** P Sasieni, J Adams, J Cuzick

**Affiliations:** 1 Cancer Research UK, Department of Epidemiology, Mathematics and Statistics, Wolfson Institute of Preventive Medicine, Charterhouse Square, London EC1M 6BQ, UK

**Keywords:** Pap smears, screening interval, case–control, cervical cytology, effectiveness, young women

## Abstract

While most experts agree that cervical screening is effective, there remains controversy over the most appropriate screening interval. Annual screening is common in North America. In England, some argue for 3-yearly screening while others believe 5-yearly screening is adequate, and the frequency varies from one part of the country to another. Screening histories of 1305 women aged 20–69 years, diagnosed with frankly invasive cervical cancer and 2532 age-matched controls were obtained from UK screening programme databases. Data were analysed in terms of time since last negative, and time since last screening smear. Five-yearly screening offers considerable protection (83%) against cancer at ages 55–69 years and even annual screening offers only modest additional protection (87%). Three-yearly screening offers additional protection (84%) over 5-yearly screening (73%) for cancers at ages 40–54 years, but is almost as good as annual screening (88%). In women aged 20–39 years, even annual screening is not as effective (76%) as 3-yearly screening in older women, and 3 years after screening cancer rates return to those in unscreened women. This calls into question the policy of having a uniform screening interval from age 20 to 64 years and stresses the value of screening in middle-aged women.

While most experts agree that cervical screening is effective, there remains controversy over the most appropriate screening interval. Annual screening is common in North America (even in women who have been screened several times previously), while 5-yearly screening is provided by some European countries. Here, we estimate the benefits of screening at different intervals and at different ages from a large population-based case–control study.

Following the relaunch of the National Cervical Screening Programmes in the United Kingdom in 1988, we initiated a protocol to monitor its effectiveness. Originally under the auspices of the National Coordinating Network and more recently with the support of the National Screening Office, we have been collecting data on the screening histories of all women from self-selected Health Authorities and Health Boards (HAs) with newly diagnosed cancer and a small control of sample women without cervical cancer. We previously reported results on 5 years of screening in 1025 women, including 348 with cancer (Sasieni *et al*, 1996). The full data set now has over 2500 women with cancer. Here we analyse data on 1305 women diagnosed with stage 1B or worse cervical cancer between the ages of 20 and 69 years, and 2532 controls with a total of approximately 40 000 women-years of screening (35 000 since 1988). Approximately 90% of cases in this report were not included in the previous report. The rationale for restricting attention to frankly invasive cancer is that ideally these should all be prevented by screening, whereas microinvasive cancers are mostly screen-detected and have an extremely good cure rate.

## MATERIALS AND METHODS

The methods have been described previously (Sasieni *et al*, 1996). The participating HAs have changed over the years due to boundary changes and changes in personnel. Areas that contributed data and the years for which they contributed are listed in the Acknowledgements. Data were collected by local coordinators as part of audit. Cases were identified from pathology laboratories and confirmed to have been resident in the HA at diagnosis. Data recorded included the date of diagnosis and, where possible, the stage and histology of the cancers. Age-matched controls, for each case, were identified from among women (not known to have had a hysterectomy) registered with a group practice (GP) in the same area. One control was selected from the same GP, the other from another GP in the same area. Screening histories (including the dates and results of all smears) were downloaded from the Exeter computer system and checked against information held by cytology laboratories. The Exeter system is used to run the screening programme and stores screening histories of all women registered with a GP. All smears in this study were prepared by conventional (as opposed to liquid-based) cytology and classified according to a version of the British Society for Clinical Cytology (BSCC) system (Johnson and Patnick, 2000).

In all analyses, a case's date of diagnosis was used as a pseudo-date of diagnosis for her matched controls, and only smears taken prior to that date were considered. All smears in teenagers were excluded because they are not part of the screening programme and are likely to identify a high-risk group. Odds ratios and their confidence intervals (CIs) were estimated by conditional logistic regression (Breslow and Day, 1980). As cervical cancer is a rare disease, odds ratios are interpreted as relative risks (RRs) and are referred to as such. Age groups refer to the age at diagnosis, not the age at which the smear was taken.

One measure of exposure is the time between the last ‘operationally’ negative smear and (pseudo-) diagnosis. An operationally negative smear is defined as a negative smear not preceded by an abnormal smear (borderline or worse) within the previous 12 months. A second measure of exposure is the time prior to diagnosis of the most recent adequate screening smear (regardless of result) ignoring all smears within 6 months of diagnosis. A screening smear is defined here to be one that was not preceded (at any time) by an abnormal smear. An adequate smear is one that was not classed as inadequate (for making a good cytological classification).

To estimate the protection from frankly invasive cervical cancer by being screened once every 3 years, we calculate the mean of the RRs for 0.5–1.5, 1.5–2.5 and 2.5–3.5 years, and similarly for 5 years. For ‘time since last negative’, the first interval is 0–1.5 years and is weighted accordingly when calculating the mean. The proportion preventable is one minus the mean RR.

## RESULTS

The database includes 2753 women with invasive cervical cancer diagnosed between 1990 and 2001 (91% between 1992 and 1998). Their age distribution (Table 1

**Table 1:** Age distribution of invasive, microinvasive and unknown stage cases from this audit compared to all UK cervical cancer registrations

	**Stage**				
**Age group (years)**	**1A**	**1B+**	**Unknown**	**Total**	**Registrations for UK 1993–1997 (%)**
<20	0 (0.0%)	1 (0.1%)	0 (0.0%)	1 (0.0%)	0.1
20–24	18 (3.3%)	*13 (0.8%)*	3 (0.6%)	34 (1.3%)	1.3
25–39	291(54.2%)	*425 (24.6%)*	157 (32.1%)	873 (31.7%)	28.7
40–54	156 (29.1%)	*481 (27.9%)*	147 (30.0%)	784 (28.5%)	26.9
55–69	61 (11.4%)	*386 (22.4%)*	93 (18.9%)	540 (19.6%)	19.8
70+	11 (2.0%)	420 (24.3%)	90 (18.4%)	521 (18.9%)	23.2
					
Total	537 (100%)	1726 (100%)	490 (100%)	2753 (100%)	100

Values in italics indicate the data analysed in the paper.

) is extremely similar to that of all cases registered in the UK for 1993–1997. Those aged over 69 (521) or under 20 (one) and those others with microinvasive cancer (526) or unknown stage (400) were excluded. Table 2

**Table 2:** Percentage of women with at least one recorded smear more than 6 months prior to (pseudo-) diagnosis by age group and disease status

**Age group (years)**	**Control**	**1B+**
20–39	83.1%	79.5%
	(721/868)	(348/438)
40–54	84.6%	67.6%
	(771/911)	(325/481)
55–69	70.7%	48.2%
	(532/753)	(186/386)
		
Total	79.9%	65.8%
	(2024/2532)	(859/1305)

provides an age breakdown of the remaining 1305 cases and their 2532 controls, and gives the proportion screened in each group. All subsequent analyses relate to the cases (aged 20–69 years with frankly invasive cervical cancer) and controls described in Table 2.

The risk of developing cervical cancer is less in the years following an operationally negative smear and the magnitude of this effect decreases (the RR increases) with increasing time since the last negative smear (Table 3

**Table 3:** Odds ratios (with 95% confidence intervals) for frankly invasive cervical cancer by time since the last operationally negative cytological smear

	**Age group (years)**		
	**20–39 (*n*=438)**	**40–54 (*n*=481)**	**55–69 (*n*=386)**
No previous negative	1.0^a^	1.0^a^	1.0^a^
*Model A* (years since last negative smear)			
0–1.5	0.24 (0.16–0.37)	0.12 (0.08–0.18)	0.13 (0.08–0.22)
1.5–2.5	0.33 (0.21–0.51)	0.14 (0.08–0.22)	0.13 (0.07–0.23)
2.5–3.5	0.67 (0.43–1.04)	0.25 (0.16–0.40)	0.15 (0.08–0.26)
3.5–4.5	1.06 (0.65–1.72)	0.30 (0.18–0.50)	0.18 (0.09–0.34)
4.5–5.5	1.40 (0.75–2.62)	0.61 (0.34–1.09)	0.28 (0.14–0.57)
5.5–6.5	1.86 (0.88–3.93)	0.72 (0.36–1.43)	0.33 (0.14–0.79)
Over 6.5	2.37 (1.16–4.85)	0.69 (0.36–1.34)	0.55 (0.27–1.10)
One negative only	1.00 (0.70–1.41)	1.31 (0.90–1.91)	1.39 (0.88–2.21)
			
*Model B* (years since last negative smear)			
0–2.9	0.28 (0.20–0.41)	0.12 (0.08–0.17)	0.13 (0.08–0.19)
3.0–4.9	1.03 (0.68–1.56)	0.39 (0.26–0.58)	0.20 (0.12–0.33)
Over 5.0	2.05 (1.20–3.49)	0.72 (0.43–1.18)	0.45 (0.25–0.81)
One negative only	1.03 (0.73–1.46)	1.32 (0.91–1.91)	1.35 (0.85–2.14)

a Baseline.

(A) Analysis by single year since last negative. B) Analysis using grouped time since last negative. Both models include a dummy variable for those with just one negative smear. *n* is the number of cases included in the analysis; there are approximately twice as many controls.

and Figure 1

**Figure 1:**
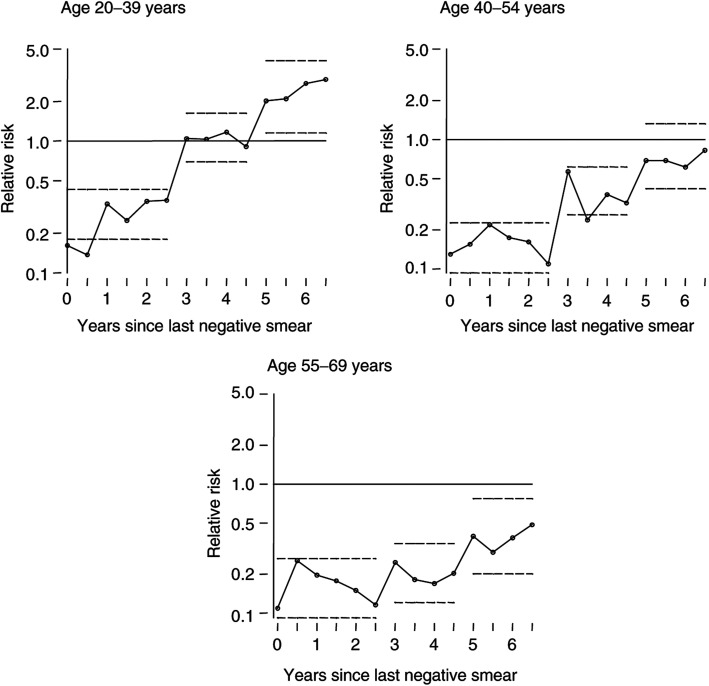
Relative risk of cervical cancer as a function of time since last operationally negative smear. The risks are calculated in 6-monthly intervals. The horizontal dotted lines mark the 95% confidence bands on the relative risks for 0–3, 3–5 and 5+ years. All estimates are relative to the risk in women who have never had a negative smear.

). The results differ between age groups. Relative to those who have never had a negative smear, women aged 40–69 years (at the end of the interval) are very unlikely (RR 0.12 and 0.13 for those aged 40–54 and 54–69 years, respectively) to develop cervical cancer within 3 years of a negative smear. Those aged 55–69 years are still moderately unlikely (RR=0.20) to develop cancer 3–5 years after a negative smear, and remain at lower risk (RR=0.45) for well over 5 years. For women aged 40–54 years, the risk increases more rapidly: RR=0.39 after 3–5 years, with close to the background risk after 5 years. In younger women, protection is weaker and even more time restricted: RR=0.24 in the first year, RR=0.28 over the first 3 years, and there is no effect 3–5 years after a negative smear (RR=1.03). In those aged 20–39 years at diagnosis, the risk more than 5 years after a negative smear is significantly greater than in those who have never had a negative smear.

In young women, having a last negative smear over 5 years ago is associated with a doubling of risk compared to women who never had a negative smear. The estimates could be confounded by demographics: women who go for regular screening may have a different underlying risk of disease than those who are only screened infrequently or those who never attend screening. Excluding the district controls and comparing cases only to their GP controls had little overall effect on the results. Similarly, excluding all smears taken before 1988 because of concern of ‘information bias’ in the preferential recording of historic smears in cases had minimal effect (results not shown).

Figure 1 illustrates the RR of cancer as a function of time since the last negative smear calculated using 6-monthly intervals. Note the substantial jumps (approximately two-fold) either side of 3 and 5 years. Further, in each age group, the estimated RR 3–3.5 years after a negative smear is greater than after 3.5–4 years.

Relative risks as a function of time since the last adequate smear (excluding smears within 6 months of diagnosis) are shown in Table 4

**Table 4:** Odds ratios for fully invasive cervical cancer by time since last adequate cytological smear ignoring all smears within 6 months of diagnosis

	**Odds ratio (95% CI)**		
	**20–39 (*n*=438)**	**40–54 (*n*=481)**	**55–69 (*n*=386)**
No adequate smear more than 6 months prior to diagnosis	1.0^a^	1.0^a^	1.0^a^
*Time since last adequate smear*^b^ *(years)*			
0.5–1.5	0.77 (0.53–1.11)	0.38 (0.26–0.54)	0.42 (0.27–0.65)
1.5–2.5	0.35 (0.23–0.54)	0.22 (0.14–0.34)	0.21 (0.12–0.36)
2.5–3.5	0.65 (0.43–0.98)	0.34 (0.23–0.50)	0.18 (0.11–0.30)
3.5–4.5	0.76 (0.47–1.23)	0.28 (0.17–0.47)	0.22 (0.12–0.38)
4.5–5.5	0.98 (0.52–1.82)	0.61 (0.37–1.01)	0.32 (0.18–0.57)
5.5–6.5	1.61 (0.77–3.38)	0.80 (0.40–1.60)	0.28 (0.13–0.60)
Over 6.5	2.05 (0.89–4.68)	1.01 (0.58–1.77)	0.48 (0.26–0.89)
			
Exactly one screening smear	1.06 (0.79–1.44)	1.36 (1.01–1.82)	2.42 (1.77–3.33)

a Baseline.

b These odds ratios are appropriate for women who have had two or more adequate smears. For women with just one screening smear, they should be multiplied by the odds ratio for ‘exactly one screening smear’. Thus, for instance, a woman aged 45 years who was last screened 4 years ago and had just one smear on record would have an odds ratio of 0.38=0.28 × 1.36. Multivariate conditional logistic regression including time since last adequate smear and an indicator for those with just one screening smear. *n* is the number of cases included in the analysis; there are approximately twice as many controls.

. Notice that (i) the RRs are generally greater than in Table 3, (ii) the risk in the period 0–1.5 years is always greater than that for 1.5–2.5 years, and (iii) there is only a modest effect of screening in those aged 20–39 years at diagnosis.

Table 5

**Table 5:** Summary percentage preventable by 3- and 5-yearly screening (these are based on the average RRs from 0–1.5, 0 to 3.5 years and 0 to 5.5 years from Tables 3 and 4)

	**20–39 years**			**40–54 years**			**55–69 years**		
**Method**	**Annual**	**3 yearly**	**5 yearly**	**Annual**	**3 yearly**	**5 yearly**	**Annual**	**3 yearly**	**5 yearly**
Last negative	76%	61%	30% (39%)	88%	84%	73%	87%	87%	83%
Last adequate		41%	30%		69%	63%		73%	73%

^*^The percentage in parentheses is obtained by replacing RRs greater than one with 1.0 when averaging.

provides a summary of the estimated proportion of cancer that could be ‘prevented’ by screening every one, 3 or 5 years. The estimates obtained using the two different methods of analysis are similar, although, as expected, the estimates based on the time since the last negative smear are generally greater. The consistent finding is that the proportion of cancer preventable by screening increases with age, but both the absolute and the relative advantage of 3- *vs* 5-yearly screening decreases with age.

## DISCUSSION

These analyses provide quantitative estimates of the benefit of different screening intervals at different ages. We have considered cancers regardless of histological type because it is not possible to set one screening interval for the prevention of squamous cell carcinoma and another for adenocarcinoma. Although cytological screening may be less effective against adeno-carcinoma, it appears to have a substantial impact (Sasieni and Adams, 2001).

It is inappropriate to compare the time since the most recent negative smear in screen-detected cases with that in randomly selected controls (IARC, 1986; Sasieni, 2001), because for a screen-detected case the time since her last negative smear will be her normal screening interval plus a short period to allow for diagnosis. It is not possible to identify which cases are screen-detected using routine data, because approximately 50% of women screened in England in the mid-1990s did not attend in response to an invitation (Department of Health, 1996). Even if GP notes were examined, it is doubtful whether one could distinguish between symptoms reported during a routine screening examination and symptoms that lead to the consultation. By considering only frankly invasive (stage 1B+) cancers, we hoped to eliminate screen-detected cancers, but in a series of 327 such cancers from southern England, 78 (24%) were classified as screen-detected (Herbert *et al*, 2001).

Screening intervals in this study were primarily either just over 3 or just over 5 years. Hence, if screen-detected cancers are included, the RR for 0–3 years will be artificially low, but it will increase substantially for 3–3.5 years. This is precisely the pattern observed (Figure 1). It is beyond the scope of this study to quantify the extent to which women with screen-detected stage 1 cancers benefited from screening.

Although we have used these RRs to estimate the absolute reduction in stage 1B+ cervical cancer that might result from 1-, 3- and 5-yearly screening, these should not be used in formal calculations of the cost per cancer prevented since negative smears do not prevent cancer. This method will overestimate the proportion of cancers prevented since not all women with abnormal smears will be prevented from developing cancer (Sasieni *et al*, 1996). Nevertheless, it does provide valuable information on the natural history of cervical precancer and of the relative benefit of different screening intervals.

Relating incidence to the time since the most recent screen estimates the extent to which a woman who has been screened recently is prevented from developing cancer. However, it is difficult to determine whether a smear was taken for screening purposes: a high proportion of cases had at least one smear in the months preceding diagnosis and many of these women had no other smears on record. For that reason, we excluded all smears taken during the 6 months immediately preceding (pseudo-) diagnosis. A period of 6 months was chosen by examining the proportions whose most recent smear was within various intervals from diagnosis (Table 6

**Table 6:** Proportion of women aged 20–69 years whose most recent smear was within different intervals

**Most recent smear**	**Control (%)**	**1B+ (%)**
⩽1 month	1.6	22.5
1–3 months	4.0	27.0
3–6 months	6.0	10.1
⩽6 months	11.6	59.6
6 months–1 year	11.3	5.1
1–6 years	54.7	15.5
>6 years	4.1	3.6
Never	18.4	16.3
		
Total	2532	1305

Time measure from date of (pseudo-) diagnosis.

). Half of all cases had a smear within 3 months of diagnosis compared to just 5% 6–12 months prior to diagnosis. In fact, the 6-month exclusion (used in Table 4) is not long enough to take account of all ‘diagnostic smears’ – the relative proportions last screened 6–12 months, compared to 1–6 years, prediagnosis is greater in cases than controls (*P*=0.002).

Another difficulty with identifying the last screening test is that once a woman has been treated for a cervical lesion, she could be put on indefinite annual follow-up. Our solution of censoring screening histories at the first abnormal smear is not ideal and it potentially introduces a small bias in favour of screening. Further, the fact that the RRs (in Table 4) are greater in the first time period than the second suggests that the 6-month exclusion is not quite sufficient and that these RRs should not be overinterpreted. Despite these caveats, we believe that this approach does provide reasonable estimates of the efficacy of 3- and 5-yearly (but not annual) screening (Table 5).

In younger women, the risk of disease in those whose last smear was more than 5.5 years ago was greater than in those who had no smears (Table 3 and 4). This suggests that those who opt out of screening altogether are at a lower underlying risk of cervical cancer than those who are screened occasionally. Opportunistic screening of women seeking contraceptive advice and those attending STD clinics could account for such a trend. What then is the appropriate baseline for estimating the RRs? We have used those with no smears, but use of those with no recent smear would have made the estimated effect of screening considerably greater in young women. The RR in women aged 20–39 years, whose most recent operationally negative smear was 3.5–4.5 years ago, was 1.06 relative to those with no such smear (Table 3). However, relative to those whose most recent negative smear was more than 6.5 years ago, it is 0.45 (=1.06/2.37). In our opinion, such adjusted RRs are inappropriate: (ignoring the effect of screening) it is more likely that those who were last screened many years ago form a high-risk subgroup than that those who are never screened are at low risk. But this needs to be tested by a larger, more detailed study in young women in which risk factors for the acquisition and persistence of HPV infection are collected along with screening histories.

## POLICY CONSIDERATIONS

Policy should be determined by balancing costs against benefits. Although there are fixed overheads, the main cost is proportional to the number of screening tests. Thus, 3-yearly screening will cost 60–66% more than 5-yearly screening. This is partly offset by not having to pay for the treatment of cancers prevented, but the financial saving is modest. The main benefits are in terms of cancers prevented and lives saved and the latter can be converted into years of life saved. There are also ‘negative benefits’ such as unnecessary treatment and anxiety caused by abnormal smears. Icelandic data show that the amount of low-grade disease (and hence, presumably, the number of women made anxious) is inversely proportional to the screening interval (Sigurdsson and Adalsteinsson, 2001). One can try to combine these factors in an overall measure of quality of life, but it is difficult to balance the low level of anxiety provoked in many women against the prevention of cancer in a few. It is also unclear whether giving one woman an extra 30 years of life is equivalent to giving an extra week to 1500 women.

Our results clearly show that cytological screening is less effective at preventing frankly invasive cervical cancer in women under the age of 40 than it is in women aged over 40 years. They also suggest that cervical cancer develops more rapidly in young women so that the incidence rate of cervical cancer 3 years after a negative smears is the same as that in unscreened women. It is possible that although screening is not very effective at preventing cancer in young women, it saves lives through early diagnosis. Indeed, in this series of 747 staged cancers in women aged 20–39 years, 41% were microinvasive.

Our results in young women differ from those reported by IARC (1986). That paper summarised the results from three cohort studies with a total of 148 cases aged under 35 years and found that the protection, relative to historical incidence rates, was similar to that seen in older women. Cases in that study were restricted to those with squamous cancer, but included stage 1A tumours (including any that were screen-detected). It is thus possible that differences in the design of the studies could explain the different results.

The RR associated with various screening intervals estimated here will help formulate policy, but there will be other considerations such as the underlying age-specific incidence rates, the numbers of years of cancer-free life gained and the age-specific rates of cytological abnormalities. Our own recommendations are given in Table 7

**Table 7:** Provisional screening recommendations for the UK

**Age group (years)**	**Frequency of cytological screening**
Under 25	Do not screen
25–49	3-yearly screening
50–64	5-yearly screening
65+	Only screen those who have not been screened since age 50

Note that these age groups are shifted by 5 years from those elsewhere in this paper to allow for the time from screening to cancer diagnosis in Tables 3–5.

. Under the age of 25 years, invasive cancer is extremely rare, but cytological abnormalities are common (Department of Health, 2001). Although lesions treated in very young women may prevent cancers from developing many years later, the results of this paper would suggest that it is enough to begin screening around age 25 – lesions that are destined to progress will still be screen-detectable and those that would regress will no longer be a source of anxiety. Nationally, only 1.7% of cervical cancer in women aged 20–69 occur under the age of 25, corresponding to an incidence rate of 2.5 per 100 000 women-years. In our study, 26 out of the 34 women with cervical cancer aged 20–24 years had a previous (operationally) negative smear, suggesting that cytology is not very sensitive for these tumours. A review of the screening histories of the 13 women with stage 1B+ cervical cancer aged 20–24 indicates that six of these cases were symptomatic, of which five were stage 1B and the other was stage 3.

In the UK, cervical cancer rates between the ages of 25 and 40 years are only slightly lower than in older women, so effective screening in this group is essential. However, most cancers still occur in older women, so resources also have to be allocated to ensure that a high proportion of women continue to be screened (albeit less frequently) at older ages.
